# Mechanical Forces between Mycobacterial Antigen 85 Complex and Fibronectin

**DOI:** 10.3390/cells9030716

**Published:** 2020-03-14

**Authors:** Albertus Viljoen, David Alsteens, Yves Dufrêne

**Affiliations:** 1 Louvain Institute of Biomolecular Science and Technology, UCLouvain, Croix du Sud, 4-5, bte L7.07.07, B-1348 Louvain-la-Neuve, Belgium; albertus.viljoen@uclouvain.be (A.V.); david.alsteens@uclouvain.be (D.A.); 2 Walloon Excellence in Life sciences and Biotechnology (WELBIO), 1300 Wavre, Belgium

**Keywords:** mycobacteria, antigen 85 complex, fibronectin, strong bonds, atomic force microscopy

## Abstract

Adhesion to extracellular matrix proteins is an important first step in host invasion, employed by many bacterial pathogens. In mycobacteria, the secreted Ag85 complex proteins, involved in the synthesis of the cell envelope, are known to bind to fibronectin (Fn) through molecular forces that are currently unknown. In this study, single-molecule force spectroscopy is used to study the strength, kinetics and thermodynamics of the Ag85-Fn interaction, focusing on the multidrug-resistant *Mycobacterium abscessus* species. Single Ag85 proteins bind Fn with a strength of ~75 pN under moderate tensile loading, which compares well with the forces reported for other Fn-binding proteins. The binding specificity is demonstrated by using free Ag85 and Fn peptides with active binding sequences. The Ag85-Fn rupture force increases with mechanical stress (i.e., loading rate) according to the Friddle–Noy–de Yoreo theory. From this model, we extract thermodynamic parameters that are in good agreement with previous affinity determinations by surface plasmon resonance. Strong bonds (up to ~500 pN) are observed under high tensile loading, which may favor strong mycobacterial attachment in the lung where cells are exposed to high shear stress or during hematogenous spread which leads to a disseminated infection. Our results provide new insight into the pleiotropic functions of an important mycobacterial virulence factor that acts as a stress-sensitive adhesin.

## 1. Introduction

Mycobacteria are important human and animal pathogens causing devastating diseases, such as tuberculosis (*Mycobacterium tuberculosis*), leprosy (*Mycobacterium leprae*), Buruli ulcer (*Mycobacterium ulcerans*), Johne’s disease (*Mycobacterium avium* subsp. *paratuberculosis*) and a myriad of opportunistic infections (atypical mycobacteria). Recently, intrinsically multidrug resistant *Mycobacteroides abscessus* (formerly *Mycobacterium abscessus*) species have drawn great attention for the difficult-to-treat infections they cause among cystic fibrosis patients who appear particularly vulnerable to them [1,2]. Also, there are reports of deadly disseminated infections even among non-cystic fibrosis patients [3,4,5,6]. Therefore, innovative therapies are needed to supplement an inadequate supply of antimycobacterial agents in a context of increasing drug resistance. One such high-potential, yet under-explored therapeutic avenue may rely on inhibition of mycobacterial adhesion to extracellular matrix proteins like fibronectin (Fn), which is important for the initial establishment and later dissemination of infection [7,8,9,10]. This requires a better understanding of mycobacterial adhesin interactions and how they respond to physical stress.

**Figure 1:**
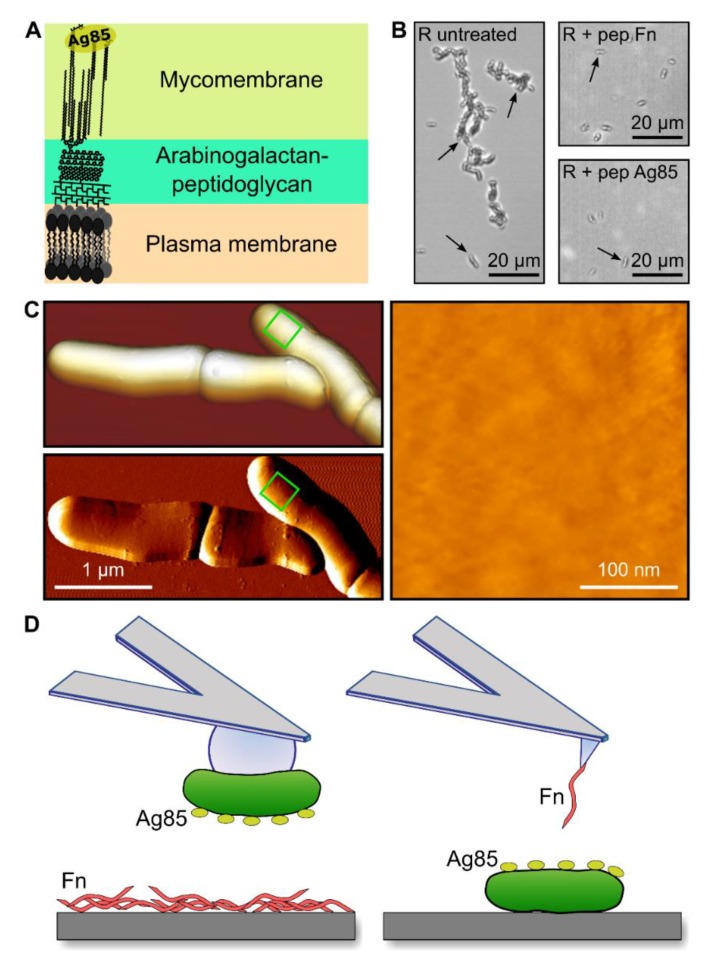
Mycomembrane-associated Ag85 is used by *M. abscessus* to bind Fn. (**A**) Secreted Ag85 is associated with the mycomembrane, which it helps produce via its mycolyltransferase activity and, where it is exposed on the bacterial surface to participate in adhesin-ligand interactions. (**B**) *M. abscessus* cells (pointed out by black arrows) attached to Fn-coated substrates (left panel), while bacterial attachment was inhibited by the addition of both Fn_pep_ (top right panel) and Ag85_pep_ (bottom right panel). At least 10 images for each condition from two independent experiments showed similar results. (**C**) Smooth surface topology observed for whole *M. abscessus* rough (R) variant cells by contact mode imaging (top left, 3D-projection of height data; bottom left, vertical deflection image) and 300 × 300 nm zoom 3rd degree-polynomial flattened height image (right panel) taken on top of one cell (green squares in the left panels indicate where the zoom was performed). Data representative of 29 images. (**D**) Cartoons depicting, on the left, the single-cell force spectroscopy and on the right, the single-molecule force spectroscopy approaches followed to study molecular force interactions between Ag85 and Fn.

In *Mycobacterium tuberculosis*, the antigen 85 complex (Ag85) consists of three paralogous proteins (Ag85A-C) that are secreted at high levels and elicit strong humoral as well as cell-mediated immune responses [11,12,13]. Although they are secreted into the extracellular milieu in large amounts, there is strong evidence that a proportion remains cell surface associated [7,14,15,16,17,18,19] where they participate via their mycolyltransferase activity in synthesis of the mycomembrane [20] (Figure 1A). Importantly, all three members of the complex were found to bind Fn indicating a role for the complex in attachment to extracellular matrix proteins, a first step in host invasion [7,8,9]. Binding of purified Ag85B to human epithelial cells was significantly reduced by siRNA-mediated Fn-depletion [21] and Ag85-Fn complexes have been identified in patients suffering from tuberculosis, further supporting a role for the Fn-Ag85 interaction during infection [22]. The amino acid residues critical to the Ag85-Fn interaction were mapped for Ag85B from *Mycobacterium kansasii* to ^98^FEWYYQSGLSV^108^, a sequence that does not show homology to other prokaryotic or eukaryotic Fn-binding proteins [23]. Conversely, the Ag85-binding fragment in Fn has the sequence ^17^SLLVSWQPPR^26^ that maps to the Fn type III_14_ module, therefore offering a unique binding site to Ag85 compared with other canonical and non-canonical microbial Fn-binding proteins that bind, respectively, to FnI_1-5_ and FnI_6_FnII_1-2_FnI_7-9_, FnIII_12_, or FNIII_9-10_ [21,24]. A robust pathophysiological mechanism involving the Fn-Ag85 interaction to establish infection remains incomplete, hampered by a lack of double or triple Ag85 knock-out mutants, which are unattainable due to the essential role of Ag85 in cell envelope synthesis [25,26].

Here we explore the interaction of Ag85 with Fn at the single molecule level. We concentrate on *M. abscessus*, an emerging, human-transmissible, multidrug-resistant pathogen causing severe lung infections in cystic fibrosis patients, often resulting in poor treatment outcomes [27,28]. Like other nontuberculous mycobacteria, *M. abscessus* presents as two different morphological forms, i.e., a smooth (S) morphotype characterized by smooth, dome-shaped, mucoid colonies and homogenously dispersed liquid cultures, and a rough (R) morphotype characterized by rough, dry, wrinkled colonies and highly aggregated liquid cultures [29]. In addition to distinct phenotypic differences in vitro, the S and R morphological distinction also has clinical relevance as the R morphotype tends to cause much worse and more persistent infections than the S type [30,31]. The *M. abscessus* genome encodes four Ag85 orthologs (MAB_0175, MAB_0176, MAB_0177 and MAB_1579). All four polypeptide sequences harbor a signal peptide for probable secretion through the Tat system, the conserved catalytic triad of amino acids required for mycolyltransferase activity as well as a sequence with high homology (>80%) to the unique-in-nature Ag85B Fn-binding sequence from *M. kansasii*, FEWYYQSGLSV [21,32,33].

## 2. Materials and Methods

### 2.1. Chemicals and Peptides

All media and chemicals used were from Sigma-Aldrich (St. Louis, MO, USA), unless specifically stated otherwise. The two peptides used in the study were from GenScript (Piscataway, NJ, USA) and had the sequences TPNSLLVSWQPPR (Fn_pep_) and AFEWYYQSGLSVI (Ag85_pep_). Fn_pep_ was dissolved in 1 × phosphate buffered saline (PBS) at 2 mg/mL and Ag85A_pep_ was dissolved in DMSO at 20 mg/mL. The final concentrations of the two peptides in blocking experiments were 0.1 mg/mL. In AFM experiments a vehicle treatment control (0.5% *v/v* DMSO) did not show any differences in the magnitudes or frequencies of Fn-Ag85 interactions.

### 2.2. Bacterial Culture Conditions

*M. abscessus* CIP104536^T^ S and R variants were cultured in Middlebrook 7H9 medium containing glycerol (0.2% *v/v*), glucose (0.2% *w/v*) and the mild nonionic detergent tyloxapol (0.025% *v/v*) to minimize aggregation of bacteria, hereafter referred to as 7H9-GGT. In order to obtain bacterial preparations consisting mainly of single cells (devoid of clumps), bacteria were first disaggregated by several passages through a fine syringe needle (26 GA), then centrifuged to remove 7H9-GGT, re-suspended in PBS, and again passed several times through a syringe needle. Finally, this suspension was filtered through a 5 µm PVDF syringe filter (Merck), allowing mainly single bacilli to pass through. Aliquots of the single-cell suspensions (OD_600_-adjusted) were frozen at −80 °C.

### 2.3. Fn-Functionalization of Surfaces and AFM Probes

In this study, we used purified human plasma Fn [34]. Gold-coated microscopy coverslips or gold-coated OMCL-TR4 AFM probes (Olympus, Tokyo, Japan) with a nominal spring constant of approximately 0.02 N/m were immersed overnight in an ethanolic solution containing 0.1 mM of 16-mercaptododecahexanoic acid and 0.9 mM of 1-mercapto-1-undecanol. Next, they were rinsed with ethanol, dried under nitrogen flow and immersed in an aqueous solution of *N*-hydroxysuccinimide (10 mg/mL) and 1-ethyl-3-(3- dimethylaminopropyl)-carbodiimide (25 mg/mL) for 30 min. Afterwards, they were rinsed with ultrapure water and placed in a 0.1 mg/mL solution of purified human plasma Fn for 1 h. Finally, they were rinsed gently with PBS and kept in PBS until experimentation. Fn-functionalized surfaces and probes were used fresh for all experiments.

### 2.4. Fn-Attachment Assay

Fn-functionalized gold surfaces were incubated, or not, in a PBS-solution of 0.1 mg/mL Fn_pep_ or Ag85_pep_ for 30 min at room temperature. At the same time an aliquot of a single cell preparation was thawed at room temperature and treated, or not, with 0.1 mg/mL of Fn_pep_ or Ag85_pep_ for 30 min. The peptide-treated or untreated Fn-surfaces were then immersed in the corresponding peptide-treated or untreated cell suspension for 1 h. Finally, they were gently rinsed by immersing them in PBS three times, before they were inverted on a large microscopy coverslip and observed with a 40× objective.

### 2.5. Single-Cell Force Spectroscopy (SCFS)

Colloidal polydopamine-wet adhesive NP-O10 probes (Bruker, Billerica, MA, USA) were prepared as previously described [35,36]. A Fn-surface (covered with a drop of PBS) was stuck to the surface of a treated polystyrene Petri dish using double-sided sticky tape. A drop of thawed single-cell bacterial suspension was transferred onto another area of the surface of the same Petri dish and the bacteria was allowed to adhere moderately for 10 min. The dish was then filled with PBS and a colloidal probe was functionalized with a single bacterial cell by gently pressing down onto a bacterium before carrying out force-distance measurements on at least two 10 × 10 µm (16 × 16 pixel) areas on the Fn-surface for each cell using a maximum exerted force of 250 pN, a contact time of 500 ms, constant approach and retraction speeds of 1 µm/s and a ramp length of 1 µm.

### 2.6. Single-Molecule Force Spectroscopy (SMFS)

A single-cell aliquot was thawed and transferred to an untreated (hydrophobic) Petri dish (Corning, Corning, NY, United States) and allowed to adhere through hydrophobic interactions to its surface for 1 h. The dish was then rinsed twice with PBS before force-distance curves were collected on 300 nm × 300 nm (32 × 32 pixels) areas on top of single bacteria using the same AFM parameters specified for SCFS and a Fn-functionalized probe (see above). To investigate the effect of mechanical stress on the Ag85-Fn interaction, force-distance curves were collected on 300 nm × 300 nm (32 × 32 pixels) areas on top of single bacteria using standard AFM parameters apart from the retraction speed, for which the following range was used: 0.5 µm/s, 1 µm/s, 2.5 µm/s, 5 µm/s and 10 µm/s.

### 2.7. AFM Data Analysis

Adhesion forces and rupture lengths were obtained from the last rupture peak. Data were analyzed using the JPK Data Processing software and statistical analyses were performed and graphs drawn with R. For dynamic force spectroscopy (DFS) analyses, pooled breaking force vs loading rate data from 13 cells were first subset into equal log-sized bins (10^2^–3.16 × 10^2^ pN/s, 3.16 × 10^2^–10^3^ pN/s, 10^3^–3.16 × 10^3^ pN/s, 3.16 × 10^3^–10^4^ pN/s, 10^4^–3.16 × 10^4^ pN/s and 3.16 × 10^4^–10^5^ pN/s). The breaking force density plot was then obtained for each bin and the Gaussian distributions identified. Then, all data from the first Gaussian distribution in each bin, representing measurements for single bond ruptures only, were pooled together and used to draw the DFS plot as well as to fit either the Bell Evans or the Friddle–Noy–De Yoreo models. Models were fit using nonlinear least squares regression and the Gauss–Newton algorithm in R. Goodness of fit was verified with the Pearson’s product-moment correlation test.

## 3. Results and Discussion

### 3.1. Studying Fn-Binding by M. abscessus Ag85 Down to the Single Molecule Level

We first tested the ability of *M. abscessus* bacteria from the rough (R) morphotype, the more virulent form, to bind to Fn immobilized on solid substrates. Optical microscopy images confirmed that the cells adhered to Fn surfaces, while addition of the peptides Fn_pep_ or Ag85_pep_ with sequences that are critical in the Ag85-Fn interaction [21,23] substantially inhibited cell adhesion (Figure 1B). This indicates that Fn-binding proteins (FnBPs), and particularly Ag85, are expressed at the cell surface. Topographic images of the bacteria (Figure 1C) revealed a regular and homogeneous surface, consistent with earlier studies on mycobacteria [37,38], and showing that live cells can be readily imaged without apparent alteration of the cell envelope. We used single-cell force spectroscopy (SCFS) to analyze the Ag85-Fn binding forces at the whole cell level (Figure 1D, left). Single bacteria were attached onto colloidal cantilevers and the forces between the cell probes and Fn-substrates were measured. Single-molecule force spectroscopy (SMFS) with Fn-modified tips enabled to quantify the strength of single bonds (Figure 1D, right).

### 3.2. Adhesion Forces between Single Bacteria and Fn

**Figure 2:**
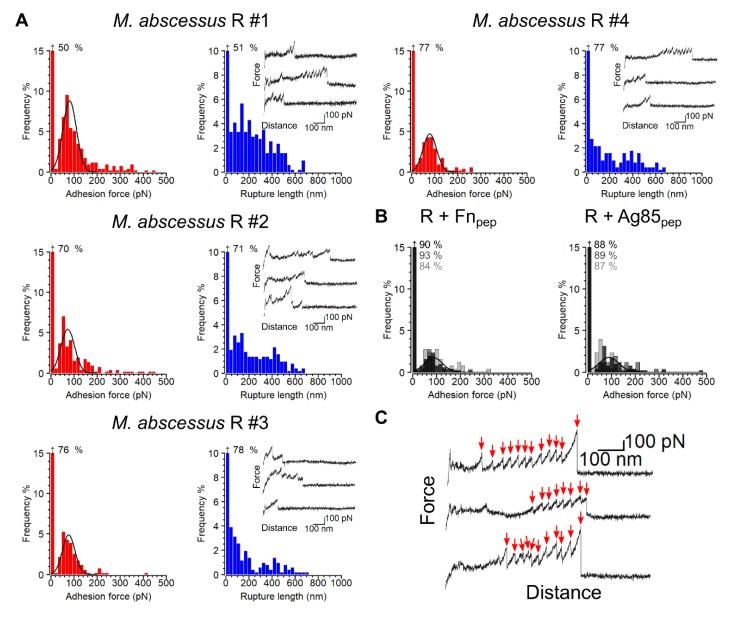
Force interactions between single *M. abscessus* R cells and Fn. (**A**) Adhesion force (left) and rupture length (right) histograms obtained for four *M. abscessus* R cells. An inset in the rupture length histograms shows three representative force-distance curves originating from the same dataset. (**B**) Injection of Fn_pep_ (left) or Ag85_pep_ (right) both resulted in significant decreases in Ag85-Fn binding frequency. Shown are overlays of three adhesion force histogram plots obtained for three different cells. (**C**) Representative force-distance curves showing sawtooth patterns of successive unbinding events, the peaks being separated by approximately 28 nm (red arrows).

We first measured the interaction forces between *M. abscessus* R bacteria and immobilized Fn by SCFS. Shown in Figure 2A, are the adhesion forces, rupture lengths and typical force curves obtained for four representative cells. Force curves (30 ± 11%) showed multiple adhesion peaks of 77 ± 29 pN magnitude (mean ± s.d.; *n* = 1051 adhesive curves from a total of 7 cells) and 271 ± 192 nm rupture lengths (*n* = 1051). To test whether these forces are associated with specific Ag85-Fn binding, two blocking experiments were performed using Fn_pep_ and Ag85_pep_. As can be seen in Figure 2B, these treatments led to a major reduction of adhesion frequency (from 30 ± 11% to 13 ± 5% and 13 ± 6%, respectively), demonstrating that Ag85 and Fn are engaged in a specific interaction. On average, the bonds ruptured at ~250 nm, but rupture distances of up to ~600 nm were also observed. Assuming that mature Ag85 comprises an average of 288 residues and that each amino acid contributes 0.36 nm to the contour length of the polypeptide chain, the fully extended protein should be ~104 nm long. This suggests that both Ag85 and Fn are being stretched upon pulling the cells away from the Fn-surfaces. Supporting this, some curves (~10% of all adhesive curves) featured sawtooth patterns (Figure 2C) with successive unbinding events (2 to 13) of 135 ± 56 pN magnitude, and peak-to-peak distances of 28 ± 9 nm, consistent with the unfolding of multiple Fn repeats. Soluble dimeric Fn has more than 50 modules with the structural β-sheet motifs FnI, FnII and FnIII. FnIII domains have been shown to unfold with forces ranging from 80 to 200 pN, leading to an increase in contour length of 28 nm for each unfolded domain [39].

The phenotypic differences between R and S *M. abscessus* variants are due to the absence and presence of glycopeptidolipids (GPLs) in R and S variants, respectively. Since, GPLs are known to be surface exposed [40], we asked whether their presence in S variant cells may have an impact on Ag85 interactions with Fn. We found that binding probability of S cells was substantially lower than that of R ones, while the magnitude of binding forces did not change significantly (Figure S1), suggesting that (i) GPL either mask surface-associated Ag85 or interfere with their interaction with Fn, and (ii) the increased Fn-binding of R variant cells plays a role in their virulence via bacterial adhesion.

### 3.3. Strength and Dynamics of Single Ag85-Fn Interactions

**Figure 3:**
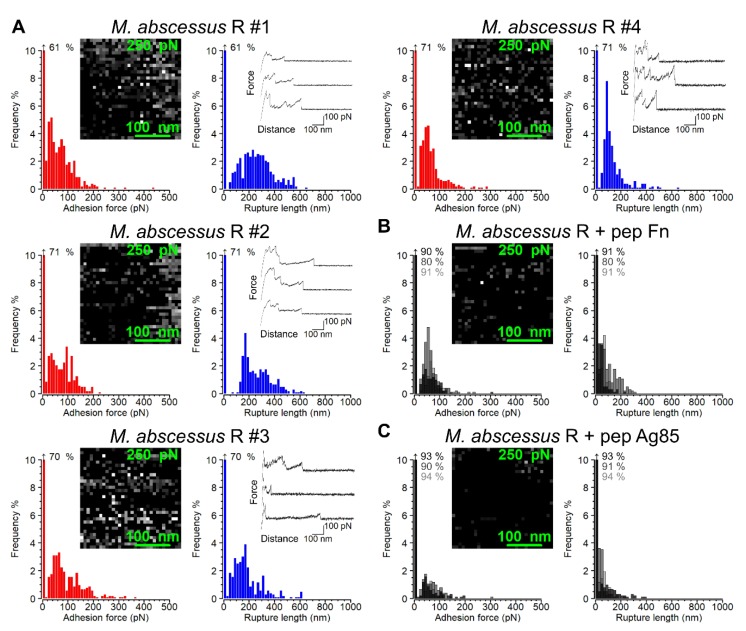
Single Fn-molecule binding to Ag85 on the surface of *M. abscessus* R cells. (**A**) Adhesion force histograms (left) and maps (inset) as well as rupture length histograms (right) and three representative force-distance curves originating from the same dataset for four *M. abscessus* R cells. (**B**) Injection of the Fn peptide resulted in significant decreases in Ag85-Fn binding events across the cell surface. Adhesion force histogram plots from three cells were overlaid. (**C**) Injection of the Ag85 peptide resulted in even greater decreases in Ag85-Fn binding events. In the adhesion maps the z-scale, depicting adhesion force in greyscale, ranges from 0 to 250 pN. Data shown are representative of at least 6 untreated or peptide-treated cells.

We then used SMFS with Fn-tips to probe single Ag85 molecules on living bacteria (Figure 3). Figure 3A shows the adhesion force and rupture length histograms with representative force curves recorded on four cells. A substantial fraction of the curves (30 ± 10%) were adhesive, with rupture peaks of 75 ± 46 pN magnitude (*n* = 1,808 adhesive force curves from 6 different cells) and rupture lengths of 137–363 nm (interquartile range, IQR), thus quite similar to the single-cell force signatures (Figure 2A). We attribute the measured adhesion forces to the detection of Ag85-Fn complexes, again based on inhibition experiments with free Fn_pep_ (Figure 3B) and Ag85_pep_ (Figure 3C), which led to major reductions in adhesion frequency (from 30 ± 10% to 19 ± 7% and 8 ± 2%, respectively) and of rupture length. Adhesion maps and frequencies (Figure 3A) demonstrated that Ag85 randomly decorates the cell surface, without any evidence of cluster formation. This is consistent with earlier analyses revealing fairly homogeneous distributions of FnBPs on *M. bovis* Bacille-Calmette-Guérin BCG cells [41]. Our ~75 pN binding forces are close to values reported for staphylococcal FnBPs [42,43,44].

**Figure 4:**
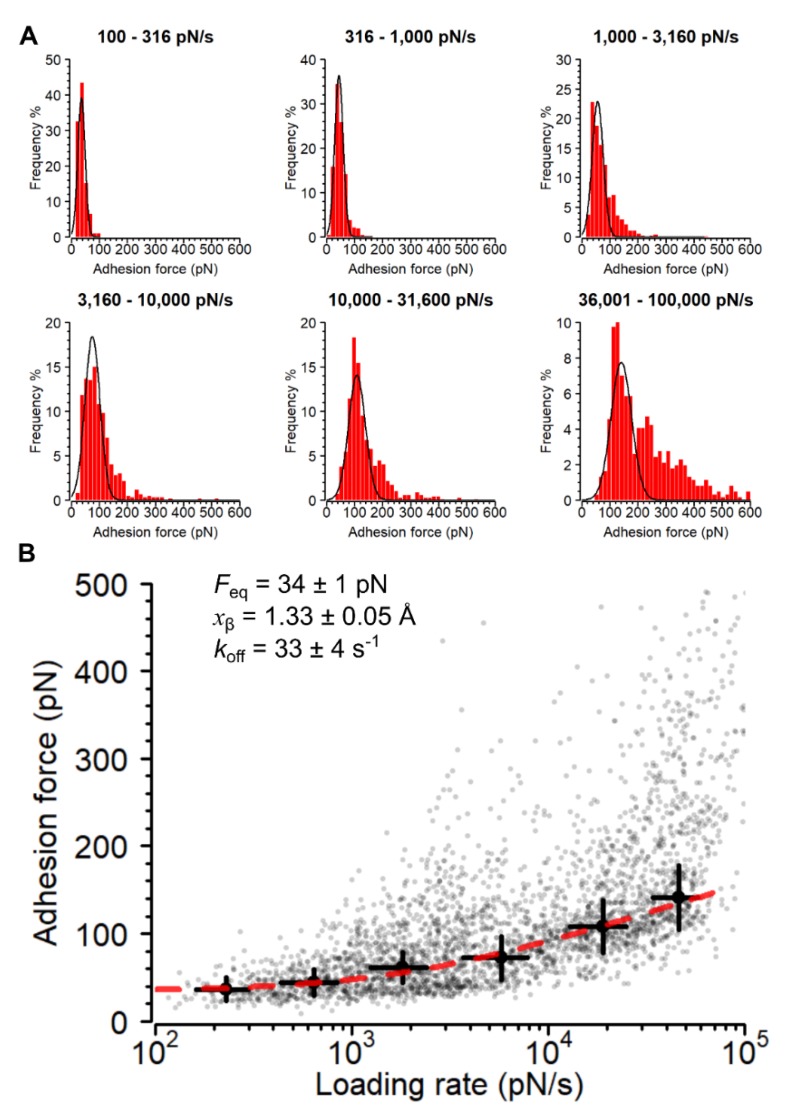
Mechanical stress strengthens the Ag85-Fn interaction. (**A**) Adhesion force histogram plots at different loading rates (*n* = 3,188 curves from 13 cells). The Gaussian distributions represent rupture events for single bonds. (**B**) Dynamic force spectroscopy plot showing Ag85-Fn binding force activation under mechanical stress. Large circles and error bars represent means and standard deviations calculated for the first Gaussian distributions at each loading rate as shown in panel A. The dashed red line represents the fit of the Friddle–Noy–de Yoreo model to the pooled data from the Gaussian bins in panel A (*n* = 2420 data points).

We next studied the Ag85-Fn interaction under increasing mechanical stress. The rupture force between receptors and ligands increases with the rate at which force is applied (loading rate, LR). While the Bell–Evans theory [45] considers a log-linear relationship between the LR and rupture force, the more recent Friddle–Noy–de Yoreo model [46] describes nonlinear trends in rupture forces, due to the reforming of a single bond at low loading rates, in the close-to-equilibrium regime. To test whether such a model would also apply to Ag85, binding forces were probed at different LR, estimated from the slope of the force vs time curves just before rupture [47]. First, we plotted the histograms of the rupture (adhesion) forces for bins of increasing loading rate, which clearly revealed that the frequency of higher adhesion forces increased with greater loading rates (Figure 4A). As can be seen in Figure 4B, the bond strength (*F*) increased nonlinearly with the LR (data obtained on 3188 adhesive curves from 13 cells). The bonds ruptured at forces ranging from 54 to 142 pN (IQR) at LRs varying from 100 to 100,000 pN/s (Figure 4B). The plot was well-fitted with the Friddle–Noy–de Yoreo equation (Pearson’s product moment correlation = 0.95, for details, see Alsteens et al. [47]). Application of Friddle–Noy–de Yoreo theory to an adhesin-ligand bond posits, that when the two molecules are pulled apart at lower LRs, they pass through an equilibrium phase where after rupture they can rebind, while a kinetic phase occurs at greater LRs where bond rupture is irreversible [46]. The fit yielded an equilibrium force (*F_eq_*) of 34 pN, a distance between bound state and transition state, *x_β_*, of 1.3 Å and a kinetic off-rate constant of dissociation, *k*_off_^0^ = 33 s^−1^, typical of single-molecular bonds. Furthermore, *F_eq_* and the effective spring constant of the system under stress can be used to calculate the thermodynamic parameter, Δ*G*_bu_, the binding free energy value, as well as the dissociation constant, *K*_d_ [47]. In order to get the most conservative estimate of these parameters for our system, we empirically determined the *k_eff_* (7 pN/nm), by taking the slope of the raw deflection vs piezo displacement for peaks yielding a breaking force of approximately 34 pN. This approach yielded a Δ*G*_bu_ of -11.9 kcal/mol and a *K*_d_ of 95 nM, a value that is in good agreement with previous affinity determinations by surface plasmon resonance of the Ag85-Fn interaction, which ranged between 30 and 70 nM [21].

Our force data are comparable with the behavior of other FnBPs. The ~75 pN binding strength measured at moderate LR is close to the forces measured for single *S. aureus* FnBPs bonds [42,43,48]. The binding strength between a synthetic peptide mimicking a single Fn-binding repeat and Fn was found to be ~100 pN [44]. Casillas-Ituarte et al. also observed nonlinear trends when plotting the rupture force vs log (LR), which they attributed to two barriers along the unbinding pathway [48]. Similar bond strengths and non-linear behaviors have been reported for the interaction of Fn with integrins [49]. However, they did not study LR of 10^5^ pN/s and their largest force measured was 160 pN. The distance from the bound state to the transition state barrier of ~1.3 Å agrees well with values determined for cell surface receptors, including FnBPs [48,49]. The dissociation rate constant, *k*_off_^0^, is comparable to that estimated for some Fn-binding repeats of FnBPA [44]. That the Ag85-Fn bond is strong at high mechanical stress, compared to other biomolecular bonds, could favor firm mycobacterial adhesion to Fn-surfaces under high shear stresses during respiratory tract infection and, importantly, during hematogenic dissemination in the case of tuberculosis [10]. Notably, it was observed that Fn-binding appeared to play an important role in attachment of *Mycobacterium bovis* BCG bacilli to bladder epithelium, where they likely encountered fluid-flow shear forces [15].

The relatively high mechanostability of the Ag85-Fn complex can be compared with that of cohesin-dockerin (Coh-Doc) complexes from cellulose-degrading enzyme networks called cellulosomes [50,51,52,53]. Single-molecule force spectroscopy and steered molecular dynamics simulations have shown that The XMod-Doc:Coh complex from *Ruminococcus avefaciens*, which mediates bacterial attachment to cellulose in the rumen, resists forces up to 750 pN at loading rates of 10^5^ pN/s [51]. The authors suggested that the high mechanostability involves stabilization of Doc by the adjacent XMod domain, and a catch bond mechanism where mechanical force increases the contact surface area of the two interacting proteins. A related complex could withstand even stronger forces, up to 1 nN [54]. Simulations revealed that this mechanical stability is achieved by a protein architecture that directs molecular deformation along paths that run perpendicular to the pulling axis. Further work is needed to elucidate the molecular origin of the force-dependent stability of the Ag85-Fn complex. Together with cellulosome and dock, lock, and latch complexes, the Ag85-Fn pair is mechanically very stable, emphasizing that mycobacteria have evolved specialized binding mechanisms to fulfil a cellular function under physical stress.

## 4. Conclusions

Mycobacterial surface proteins play an important role in guiding bacterial-host interactions (for an overview please refer to the following reviews on the topic [55,56,57]). Among these, FnBPs support mycobacterial adhesion to the respiratory mucosa via the extracellular matrix protein Fn, an interaction that is highly conserved in mycobacteria [58]. Fn-mediated adhesion has been shown for various mycobacterial species [59,60,61,62,63,64,65,66], yet the molecular forces involved are poorly understood. Elucidating the molecular basis of Fn-binding in mycobacteria is key to understanding the initial steps leading to mycobacterial infections as well as later steps of infection leading to dissemination and offers promising prospects for innovative antibacterial therapies [10].

Single Ag85 proteins bind Fn with a strength of ~75 pN under moderate mechanical stress, which agrees reasonably well with the forces reported for other Fn-binding proteins. Use of free Ag85 and Fn peptides with active binding sequences demonstrates the specificity of the interaction. The rupture force of the complex increases with tensile loading (LR) following the Friddle–Noy–de Yoreo model, which provide thermodynamic parameters that are in excellent agreement with previous affinity determinations by surface plasmon resonance. Strong adhesion (~500 pN) is observed at high tensile loading which might be of biological relevance as mycobacteria are exposed to shear stress during various stages of infection. Traditionally, bacterial adhesion is investigated under static conditions. However, many pathogenic bacteria are exposed to shear forces [67], which can largely influence binding interactions. Adhesion of *Staphylococcus aureus* to Fn differed depending on whether bacteria were incubated under static versus flow conditions [68,69,70]. Mycobacteria are exposed to a wide range of shear forces in the lung. Consistent with this, *M. tuberculosis* strains bound best to immobilized human Fn and surfactant protein A under fluid shear conditions, simulating physiological conditions within the lung [71]. It was proposed that shear conditions may reduce nonspecific binding interactions that take place under static conditions and thus, select for high-affinity binding interactions. So, we postulate that shear stress in the alveolar epithelium may favor strong Ag85-Fn binding. In the context of cystic fibrosis, where *M. abscessus* infection is of particular concern, alterations in the periciliary liquid sensitive to airflow shear was implicated in a mechanism that underlies an increased vulnerability to bacterial infections [72]. As highly homologous Ag85 orthologs are found in all mycobacteria, our conclusions on the Ag85-Fn interaction can be extended to mycobacteria in general. Perhaps mycobacteria have evolved such strong force-dependence in Ag85 to help the bacteria resist physical stress during host colonization. Various small molecules that bind to Ag85 and inhibit their mycolyltransferase activity have been identified and the Ag85 complex is considered as a potential drug target [73,74,75,76,77,78,79,80]. Ensuing studies should investigate the potential of Ag85 inhibitors in the Ag85-Fn interaction. This strategy may lead to antimycobacterial agents acting on the pleiotropic functions of the Ag85 virulence factors and may open new and more efficient avenues to treat infections with these pathogens.

## Supplementary Materials

The following are available online at https://www.mdpi.com/2073-4409/9/3/716/s1, Figure S1: Ag85-Fn force interactions in *M. abscessus* S cells.
