# Supplementary material for: Mechanical Forces between Mycobacterial Antigen 85 Complex and Fibronectin
Source: Cells. 2020 Mar 14;9(3):716. doi: 10.3390/cells9030716 (PMC7140604; doi:10.3390/cells9030716)
Supplement: Supplementary file 1 [file cells-09-00716-s001.pdf]

cells MDPI

Article

Mechanical Forces between Mycobacterial Antigen 85 Complex and Fibronectin

Albertus Viljoen [1], David Alsteens [1,2] and Yves F. Dufrêne [1,2,*]

[1] Louvain Institute of Biomolecular Science and Technology, UCLouvain, Croix du Sud, 4-5, bte L7.07.07, B-1348 Louvain-la-Neuve, Belgium; albertus.viljoen@uclouvain.be (A.V.); david.alsteens@uclouvain.be (D.A.)
[2] Walloon Excellence in Life sciences and Biotechnology (WELBIO), Belgium
* Correspondence: yves.dufrene@uclouvain.be

Supplementary Data

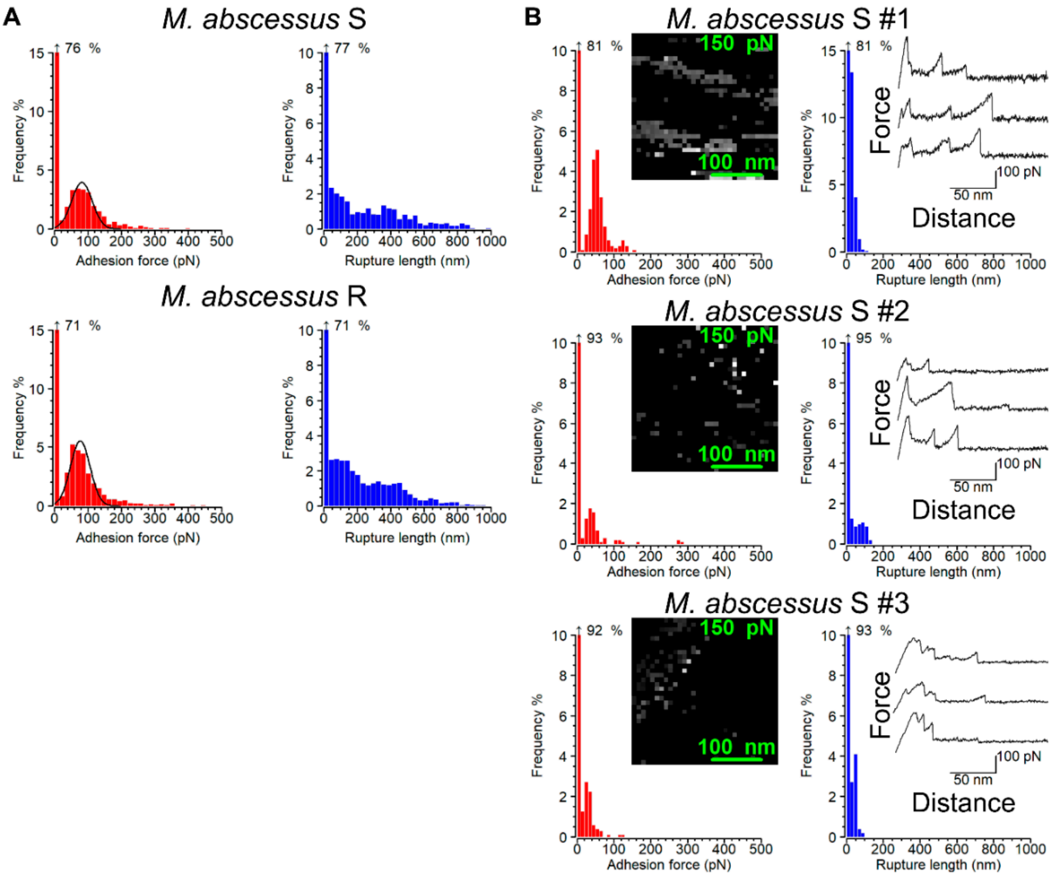

Figure S1. Ag85-Fn force interactions in *M. abscessus* S cells. (A) Adhesion force (left) and rupture length histograms (right) of single bacterium-Fn interactions shows moderately lower binding frequencies for S variant cells (top panel) compared to R variant cells (bottom panel). Histograms were drawn from the combined force-distance curve data from 7 cells of each variant. (B) Single Fn-molecule and Ag85 interactions on top of *M. abscessus* S variant cells occur significantly less frequently compared with *M. abscessus* R variant cells. Shown is adhesion force histograms (left) and maps (inset) as well as rupture length histograms (right) and three representative force-distance curves originating from the same dataset for three *M. abscessus* S cells from a total of 5 cells showing a similar trend.
